# Editorial: The interconnection between epigenetic modifications and the tumor microenvironment

**DOI:** 10.3389/fonc.2023.1166676

**Published:** 2023-04-05

**Authors:** Siyuan He, Feng Jiang

**Affiliations:** ^1^ School of Basic Medicine, Youjiang Medical University for Nationalities, Baise, China; ^2^ Department of Neonatology, Obstetrics and Gynecology Hospital of Fudan University, Shanghai, China

**Keywords:** epigenetic modification, tumor microenvironment, immunotherapy, cancer biomarkers, oncology

In the 1940s, Conrad Waddington coined the term ‘epigenetics’ to explain how the same combination of genes could produce different phenotypes in certain specific environments throughout animal development. The term epigenetics was later embraced by a wider range of disciplines and expanded to include the study of covalent and non-covalent changes in DNA and histones, as well as general alterations in chromatin structure in any biological or pathological process. For example, DNA methylation, post-translational changes in histones, chromatin remodeling, and the effects of non-coding RNAs on ribosome structure all fall within the field of epigenetic research. These epigenetic modifications translate environmental input signals into different gene combinations, allowing a limited number of transcription factors (TFs) to produce more diverse transcriptional patterns. The expression levels and biological activities of enzymes and regulators involved in epigenetic modifications may also be altered by environmental signals. Such heritable epigenetic changes with intertwined DNA/RNA/protein linkages provide a basis for studying environmental adaptations at the cellular level.

The tumor microenvironment (TME) is composed of cellular and non-cellular components, including stromal cells, immune cells, and chemokines (1). The biological importance of the TME as a response platform regulating various aspects of tumor initiation, development, metastatic progression, altered immune response, fulminant disease, and cancer recurrence is undeniable and constantly confirmed, as highlighted by numerous studies. In addition, as epigenetic alterations are associated with the control of the TME, DNA methylation may influence cancer growth by regulating immune infiltration and immune checkpoints of the TME (2, 3). In addition, histone acetylation may attenuate the immune destructive potential of the TME and promote tumor development. The main role of RNA modifications in tumor formation is to regulate angiogenesis, immune activity, and the infiltration of immune cells into the TME (4). ncRNAs released by certain cells in the TME are thought to influence the behavior of cancer cells, including invasion, metastasis, and treatment resistance (5). Furthermore, ncRNAs in tumor cells may be implicated in the immune regulation of the TME and promote tumor formation. In conclusion, epigenetic alterations hold therapeutic promise in controlling elements of the tumor microenvironment and may be a target for cancer therapy.

The objective of the Research Topic titled “*The interconnection between epigenetic modifications and the tumor microenvironment*” is to discuss recent advances in the interaction between epigenetic modifications and the tumor microenvironment and to identify potential prognostic markers and specific components that may affect the efficacy of immunotherapy and other tumor treatments. Ultimately, a total of 11 papers, contributed by more than 60 authors as experts in the field, were accepted in 30 submissions, providing new comprehensive insights for future cancer therapies.

Han et al. identified prognostic genes significantly associated with metabolic changes in hepatocellular subpopulations at the single-cell level and examined the heterogeneity of the subpopulation and its interrelationship with other cells in the tumor microenvironment. A prognostic model for predicting overall survival (OS) in patients with hepatocellular carcinoma was established, validated, and found to show good predictive ability. In addition, differences in chemosensitivity between high- and low-risk groups were assessed and five drugs were focused on that could potentially reverse the risk score.

Kahlert et al.’s original research established COL10A1, a short-chain protein belonging to the collagen family, an important component of the stromal extracellular matrix, as a diagnostic marker to predict the development of colorectal cancer, expanding on previous studies on this protein. The authors found that the abundance of COL10A1 in CRC tissue predicts the metastatic and immunogenic potential of CRC and that COL10A1 transcription may mediate the interaction between tumor cells and the stromal microenvironment.

Cui et al. investigated the function of cuproptosis-related lncRNAs in colorectal cancer (COAD). They identified six cuproptosis-associated prognostic lncRNAs in COAD and constructed a prognostic model based on cuproptosis-associated lncRNAs, providing new insight into the risk classification and possible biomarkers for patients with colorectal cancer. Analysis of the immune microenvironment, mutations, and sensitivity to chemotherapy suggests that this signature may serve as a reference for immunotherapeutic and chemotherapeutic approaches.

Yu et al. constructed a pyroptosis-related lncRNA prognostic model for predicting prostate cancer using a machine-learning approach. The researchers explored the association between the prognostic model and patients’ clinical characteristics, immune environment, immune checkpoints, gene mutations, and drug sensitivity, and constructed diagnostic and prognostic biomarkers for prostate cancer. *In vitro* experiments showed that silencing lncRNA AC005253.1 affected the expression of the AIM2 gene in prostate cancer and inhibited the proliferation, migration, and invasion of DU145 and PC-3 cells. In addition, silencing of AC005253.1 promoted the expression of pyroptosis inflammasome AIM2, and the pyroptosis-related gene AC005253.1 may be a valuable oncogene related to the prognosis of prostate cancer.

Xu et al. discussed the mechanism of MARCH1 in lung adenocarcinoma (LUAD). As a member of the E3 family, E3s were dysregulated in LUAD and were positively correlated with most immunological features, suggesting that MARCH1 may activate inflammatory TME in LUAD. Patients with LUAD with reduced MARCH1 expression had a poorer prognosis and were not sensitive to immune checkpoint inhibitors. In pan-cancer studies, MARCH1 was associated with most immunological features, suggesting that MARCH1 may be a new and promising biomarker as an indication of the immune status and effectiveness of immunotherapy in patients with LUAD.

Zeng et al. used a computational algorithm to screen out the fatty acid metabolism (FAM)-related genes associated with cervical cancer (CC) from the public databases. The FAM model (PLCB4, FBLN5, TSPAN8, CST6, and SERPINB7) risk score was an independent factor affecting the prognosis of patients with cervical cancer. This model had a high prognostic value, meaning that the FAM-related genes can be used as prognostic markers and potential immunotherapy targets for patients with CC.

The abovementioned Research Topic “*The interconnection between epigenetic modifications and the tumor microenvironment*” gathers studies focusing on the discovery of interactions between epigenetic modifications and the tumor microenvironment and the mechanisms of epigenetic modifications in immunotherapy against cancer. Several prognostic and predictive models have also been constructed that are useful for clinicians. It is hoped that this Research Topic will contribute to the understanding of the mechanisms of tumor development and provide new and broader insights into future cancer treatment.

## Author contributions

All authors listed have made a substantial, direct, and intellectual contribution to the work and approved it for publication.
